# Biomarkers and Their Association with Kidney Scarring After the First Episode of Febrile Urinary Tract Infection or Vesicoureteral Reflux in Pediatric Patients

**DOI:** 10.3390/medicina62050811

**Published:** 2026-04-24

**Authors:** Nikolaos Gkiourtzis, Panagiota Michou, Anastasia Stoimeni, Konstantinos Cheirakis, Vera Karatisidou, Theopisti Vasileiadou, Vasileios Liakos, Charalampos Antachopoulos, Kali Makedou, Nikoleta Printza, Despoina Tramma

**Affiliations:** 1 4th Department of Pediatrics, “G. Papageorgiou” General Hospital, School of Medicine, Department of Health Sciences, Aristotle University of Thessaloniki, 54124 Thessaloniki, Greece; anastoim@gmail.com (A.S.); kostxeir@hotmail.com (K.C.); vkaratisidou@gmail.com (V.K.); pistivas1951995@gmail.com (T.V.); dtramma@auth.gr (D.T.); 2 Pediatric Department, Saint Luke’s Private Clinic, 55236 Thessaloniki, Greece; giotamichou4@gmail.com; 3 3rd Department of Pediatrics, Ippokrateio General Hospital, School of Medicine, Department of Health Sciences, Aristotle University of Thessaloniki, 54124 Thessaloniki, Greece; viliakos@auth.gr (V.L.); antachop@auth.gr (C.A.); 4 Laboratory of Biochemistry, AHEPA University Hospital, School of Medicine, Department of Health Sciences, Aristotle University of Thessaloniki, 54124 Thessaloniki, Greece; kalimakedou@gmail.com; 5 1st Department of Pediatrics, Ippokrateio General Hospital, School of Medicine, Department of Health Sciences, Aristotle University of Thessaloniki, 54124 Thessaloniki, Greece; nprintza@gmail.com

**Keywords:** febrile urinary tract infection, procalcitonin, C-reactive protein, white blood cells, kidney scarring, vesicoureteral reflux, children

## Abstract

*Background and Objectives*: Children with febrile urinary tract infections (fUTIs) may be at risk for kidney scarring. Inflammatory biomarkers may predict the risk of scarring. The aim of this study was to investigate the predictive role of PCT, CRP and other markers in scarring in pediatric patients with first fUTI. *Materials and Methods*: The study was in accordance with the institution’s ethics committee (No. 108/2023). Included patients underwent a kidney–ureter–cyst ultrasound (US). The primary outcomes of this study were the associations of procalcitonin (PCT), C-reactive protein (CRP), and white blood cells (WBC) with kidney scarring. The secondary outcomes were the associations of PCT, CRP, and WBC with vesicoureteral reflux (VUR). A *p* < 0.05 was considered statistically significant. *Results*: Sixty-five pediatric patients (1–16 years of age) with a first fUTI, from February 2023 to January 2025, were included. Sixteen patients had VUR, and thirteen patients developed kidney scarring. C-reactive protein was significantly elevated in the VUR group (*p* = 0.026). In a series of logistic regression analyses, abnormal US findings and severe VUR were associated with scarring (*p* = 0.009 and *p* = 0.016, accordingly). The optimal cut-off value for PCT in predicting scarring was calculated as 6.05 ng/mL (sensitivity: 36% and specificity: 97%), and for CRP as 3.62 mg/dL (sensitivity: 63.6% and specificity: 62.1%). *Conclusions*: This study showed a significant difference in CRP levels between the groups with and without VUR. Abnormal US findings and severe VUR were the most significant predictors of kidney scarring. Significant difference was not reached in PCT and WBC levels between the groups with and without VUR or with and without kidney scarring. The small sample size may have influenced the study’s outcomes.

## 1. Introduction

Febrile urinary tract infection (fUTI) is among the most frequent bacterial infections in the pediatric population, particularly affecting infants and young children [1,2]. Children with fUTI, especially those who have a history of vesicoureteral reflux (VUR), are at increased risk for complications such as kidney scarring [3]. Recurrent urinary tract infections (rUTIs) are notably more common in children with VUR [4]. The risk of kidney scarring is estimated at 30% after three episodes of fUTI [2].

A comprehensive cohort study of children with first episodes of fUTI revealed that males are more likely to present with dysplastic or hypoplastic kidneys—with or without accompanying VUR—whereas females are more commonly affected by kidney scarring associated with rUTI episodes [5]. Technetium-99m dimercaptosuccinic acid (DMSA) scan at 4–6 months is the gold standard for kidney scarring detection, with over 90% sensitivity and specificity [6,7,8]. Long-term complications, such as proteinuria, hypertension or impaired kidney function, may follow [1].

Procalcitonin (PCT) is a 116-amino acid residue that can adequately distinguish bacterial infections, such as fUTI, from viral infections based on its serum levels, as it is not elevated in viral infections [9]. In contrast, C-reactive protein (CRP) lacks the specificity to accurately distinguish between bacterial and viral infections. Our recent meta-analysis revealed that PCT was higher in pediatric patients with scarring after an episode of fUTI compared to those without scarring [10]. Additionally, elevated levels of CRP might have a role in predicting kidney scarring after fUTI in children [11,12]. We conducted this study to investigate the predictive role of PCT, CRP, and other markers in kidney scarring detected by DMSA scan in pediatric patients with a first fUTI.

## 2. Materials and Methods

**Figure 1:**
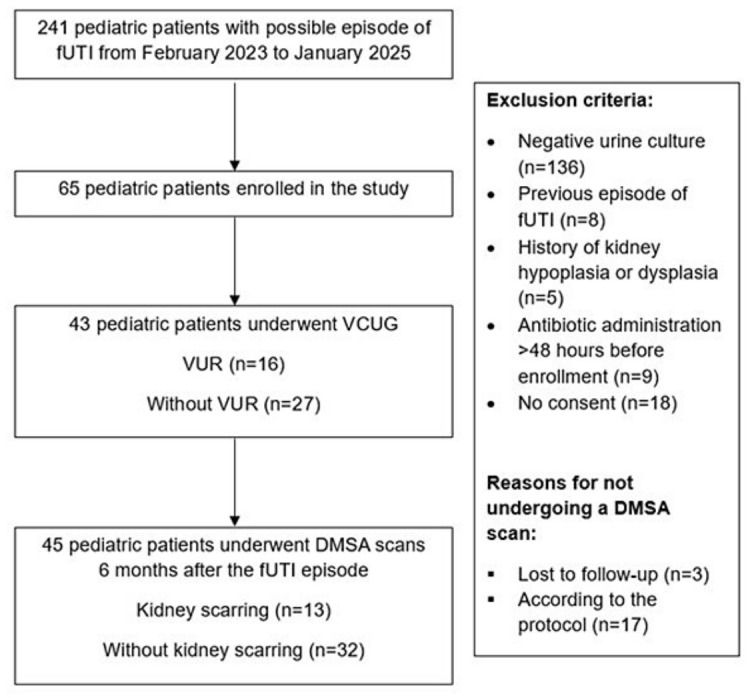
Flow chart of the prospective cohort study.

A prespecified protocol was approved by the Aristotle University of Thessaloniki (AUTH) Assembly held on 30 May 2023 (Registration Number: 5057). Sixty-five pediatric patients with a first episode of fUTI, from February 2023 to January 2025, were included in this study (Figure 1). All eligible individuals were aged from 1 day to 16 years. Patients who were hospitalized in four pediatric departments (1st, 3rd and 4th Departments of Pediatrics, AUTH, Greece and Saint Luke’s Private Clinic, Thessaloniki, Greece) and met the inclusion criteria were enrolled in the study.

### 2.1. Inclusion Criteria

The enrollment criteria were: pediatric patients under the age of 16 years old with suspected first episode of fUTI or any episode of fUTI with a previous normal DMSA scan, hospitalized in a pediatric department; body temperature ≥37.5 °C; positive urine culture collected either by the midstream clean-void technique (≥105 CFU/mL) if there was bladder control (toilet-trained children), or by suprapubic aspiration (any bacterial growth) or bladder catheterization (≥103 CFU/mL) if there was no bladder control (nontoilet-trained infants); and indicative symptoms of fUTI (vomiting, poor feeding, costovertebral angle tenderness, malaise, etc.). Following a combination of NICE [13], AAP [14], and Swedish [15] guidelines, all pediatric patients with a first episode of fUTI underwent an acute-phase kidney–ureter–cyst ultrasound (US). If there was a history of rUTI (with a previous normal DMSA scan), abnormal ultrasonographic findings, a non-*Escherichia coli* infection, or delayed treatment response (>48 h), voiding cystourethrography (VCUG) and a late DMSA scan (≥6 months) were recommended in hospitalized patients. While previously known major congenital anomalies, such as kidney dysplasia and hypoplasia, were excluded, other structural abnormalities were assessed through acute-phase ultrasound and analyzed as potential predictors rather than exclusion criteria.

### 2.2. Primary Outcomes

The primary outcomes of the study were the association of PCT, CRP, and white blood cells (WBCs) with kidney scarring, comparing the groups with and without kidney scars.

### 2.3. Secondary Outcomes

The secondary outcomes were the association of PCT, CRP, and WBC with VUR, comparing the groups with and without VUR. Additionally, the sensitivity and specificity of PCT and CRP in kidney scar detection were estimated.

### 2.4. Imaging

Following a combination of NICE [13], AAP [14], and Swedish [15] guidelines, all pediatric patients with a first episode of fUTI underwent a kidney–ureter–cyst US. If there was a history of rUTI (with a previous normal DMSA scan), abnormal ultrasonographic findings, a non-*Escherichia coli* infection, or delayed treatment response (>48 h), a voiding cystourethrography (VCUG) and a DMSA scan were recommended in hospitalized patients. VCUG was performed based on clinical indications rather than systematically in all patients, which may have led to underdiagnosis of VUR and potential bias in assessing its association with kidney scarring.

Kidney US was performed in all patients during the acute phase of fUTI. Voiding cystourethrography was performed in participants who met the above criteria after treatment of the fUTI episode, before discharge, and no later than eight weeks after the acute infection, following a negative urine culture. Finally, a late DMSA scan was performed in participants who met the above criteria at least six months after the fUTI episode [13]. Kidney scarring was defined according to the guidelines of the European Association of Nuclear Medicine [16].

### 2.5. Treatment

All children were hospitalized and treated with empirical antibiotics (combination of ampicillin and amikacin, combination of amoxicillin–clavulanic acid and amikacin, or second- or third-generation cephalosporins) according to local guidelines for 10 days. The antibiotic regimen was adjusted according to the culture-proven organism.

### 2.6. Sampling

All serum and urine samples were collected on the same day that antibiotic treatment was initiated. White blood cell count, CRP and PCT levels were obtained. In addition, extra blood and urine samples were collected and stored at −32 °C. Quantitative estimation of PCT levels were performed using a sandwich immunoluminometric method, with a detection limit of >0.08 ng/mL. Additionally, CRP levels were estimated using an enzyme-linked immunosorbent assay method (detection limit 0.5 mg/dL).

### 2.7. Statistical Analysis

Data were analyzed using Microsoft Excel for Windows 11 and R software (version 4.3.2). Normally distributed continuous variables are described as mean ± standard deviation (SD), while skewed variables are presented as median ± interquartile range (IQR). Independent sample *t*-tests were applied to examine differences between groups for normally distributed variables, whereas the non-parametric Mann–Whitney test was used for skewed variables. Qualitative variables were compared by the Chi-squared test or Fisher’s exact test where needed, and results were expressed as percentages. After univariate logistic analyses, multivariate logistic regression analysis, utilizing backward conditional elimination with entry criteria set at 0.1, was employed to identify significant predictors of the selected dependent variables. Finally, every available marker was compared in terms of discriminability for the diagnosis of kidney scarring using receiver operating characteristic (ROC) curves. A *p* < 0.05 was considered statistically significant.

### 2.8. Ethical Approval

All procedures performed in studies involving human participants were in accordance with the ethical standards of the institution’s ethics committee (No. 108/2023), at which the study was conducted, and with the Declaration of Helsinki (1964) and its later amendments or comparable ethical standards. Before study enrollment, informed consent was obtained from the parents or legal guardians of the participants.

## 3. Results

### 3.1. Patient Characteristics

Among 241 pediatric patients with possible episodes of fUTI, 65 individuals were included in the study (Figure 1). The median age of participants with a first episode of fUTI was 4.00 months [2.00–8.00] (Table 1). Thirty-seven non-circumcised males made up 56.9% of all participants, and fifty-eight children (89.2%) were below 12 months of age. The median duration (days) of fever before the initiation of antibiotic treatment was 2.00 [1.00–3.00].

*Escherichia coli* was cultured as a single pathogen in twenty-nine children (44.6%), *Klebsiella pneumoniae* in eight (12.3%), extended-spectrum beta-lactamases (ESBL)-producing *Escherichia coli* in four (6.1%), *Enterococcus faecalis* in four (6.1%), *Pseudomonas aeruginosa* in three (4.6%), Proteus mirabilis in three (4.6%), and other pathogens in 21.5%.

Among children with fUTI, six participants had mild hydronephrosis (9.2%) on ultrasonography, four had moderate hydronephrosis (6.1%) and two had severe hydronephrosis (3.1%).

Only two patients relapsed during the follow-up period. Of 65 children with a first episode of fUTI, 45 individuals (69.2%) underwent a late-phase DMSA scan (at least six months after the first episode of fUTI). Kidney scarring was detected in 13 patients (28.9%). Seven patients developed mild scarring (53.8%), and six patients developed moderate scarring (46.2%) [17].

### 3.2. Infants Under 12 Months

The mean age of infants with a first fUTI was 4.41 months (2.87). The male-to-female ratio was 36:22. Among these patients, 19% had a degree of hydronephrosis, 25.9% had a degree of VUR, and 20.7% had a degree of scarring on DMSA scan six months after the episode of fUTI. *Escherichia coli* was the most common microorganism cultured in urine cultures (37.9%).

### 3.3. Patients with and Without VUR

Among 65 patients with a first fUTI, 43 underwent VCUG and 16 of them had VUR (Table 2). No statistically significant differences were found in age, sex, or fever duration between the groups with and without VUR. The median age was slightly higher in the VUR group (4.5 vs. 3.0 months, *p* = 0.56), and the proportion of males was lower (*p* = 0.16), though neither reached statistical significance. Inflammatory markers, including PCT and WBC, did not differ significantly between groups (*p* = 0.21 and *p* = 0.74, respectively). However, CRP levels were significantly elevated in the VUR group compared to those without VUR (*p* = 0.026). Obviously, kidney scarring was more frequently present in patients with VUR (*p* = 0.03). Finally, no significant differences were found in prematurity or abnormal US findings between the groups.

### 3.4. Patients With and Without Kidney Scarring

**Figure 2:**
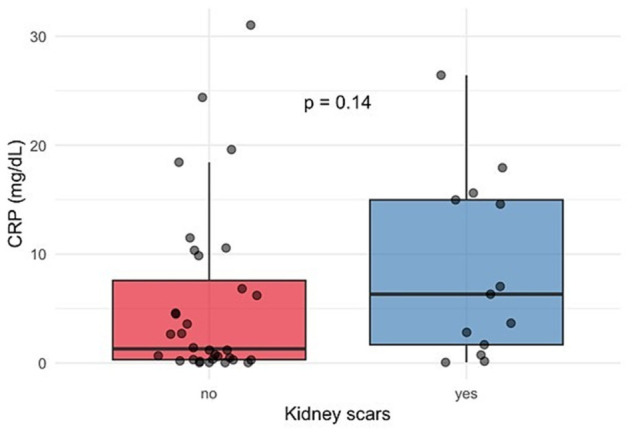
Association of CRP levels in pediatric patients with and without kidney scarring after the first episode of fUTI (box plot); CRP: C-reactive protein; fUTI: febrile urinary tract infection.

**Figure 3:**
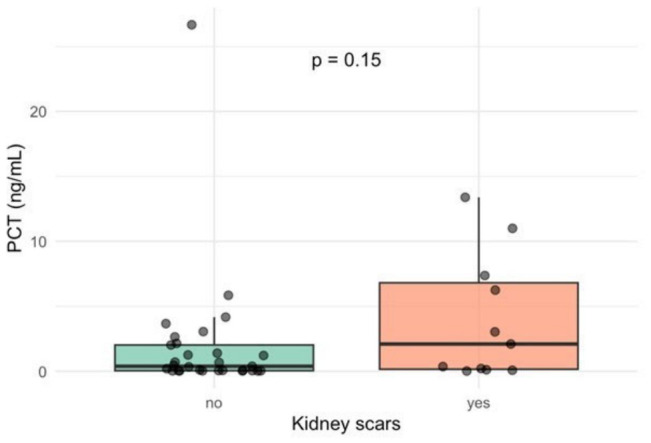
Association of PCT levels in pediatric patients with and without kidney scarring after the first episode of fUTI (box plot); fUTI: febrile urinary tract infection; PCT: procalcitonin.

Forty-five children with a first fUTI episode underwent a DMSA scan six months after the episode of fUTI, and thirteen of them developed scarring (Table 3). A comparison between children with and without kidney scarring after fUTI showed no significant differences in age, sex, or fever duration. The median age was similar between groups (*p* = 0.89), and the proportion of males was slightly higher in the scarring group (*p* = 0.64). Procalcitonin and CRP were elevated in the scarring group, though differences did not reach statistical significance (PCT: 2.10 vs. 0.39 ng/mL, *p* = 0.15; CRP: 8.62 vs. 1.30 mg/dL, *p* = 0.14) (Figure 2 and Figure 3). Additionally, WBC counts were also comparable between groups (*p* = 0.46). As expected, abnormal US findings were more common in children with kidney scars (*p* = 0.008).

### 3.5. Logistic Regression

In a series of univariate logistic regression analyses examining predictors of kidney scarring in children with fUTI, several clinical variables were evaluated. Sex was not a significant predictor, with females showing no significant difference compared to males (OR = 0.57, *p* = 0.42). Age also did not significantly influence the odds of scarring (OR = 0.99, *p* = 0.76). Similarly, PCT and CRP were not statistically significant predictors (*p* = 0.29 and *p* = 0.241, respectively), despite suggestive trends. In contrast, abnormal acute-phase US findings were strongly associated with the presence of kidney scars (OR = 6.75, *p* = 0.009), indicating a significant risk factor. Furthermore, VUR severity was also associated with scarring, with severe VUR demonstrating a significant increase in the odds of scarring (OR = 6.80, *p* = 0.016). Additionally, a multivariable logistic regression model was developed to identify independent predictors of kidney scarring (Table 4). Variables with a univariate *p*-value less than 0.1 were considered for inclusion in the model, while PCT and CRP were also included based on their established clinical relevance as markers of infection severity. Abnormal US was associated with kidney scarring (OR = 6.99, 95% CI: 1.24–53.53, *p* = 0.037), and severe VUR showed a positive association (OR = 3.72, 95% CI: 0.64–26.37), although this was not statistically significant (*p* = 0.15). PCT and CRP were not significant predictors in this model. Backward stepwise selection supported a reduced model including US, VUR, and PCT (final AIC = 41.98), underscoring the predictive value of imaging findings and the clinical relevance of inflammatory markers. In the reduced model, abnormal US findings were a significant predictor of kidney scarring (OR = 6.39, 95% CI: 1.22–44.18, *p* = 0.037). No multicollinearity was detected. Model fit was acceptable, with a McFadden pseudo-R^2^ of 0.215.

**Figure 4:**
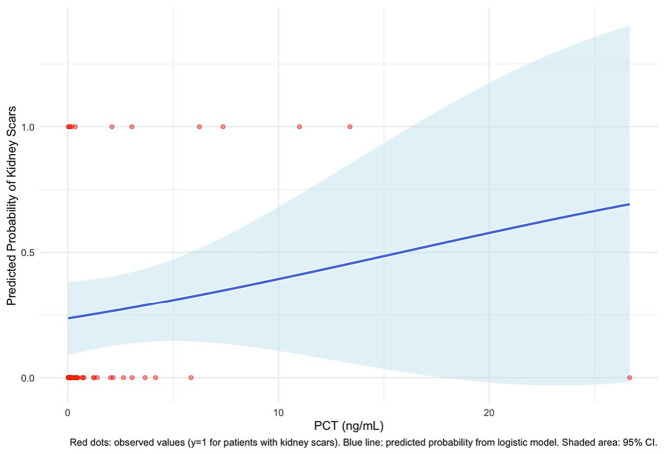
Association between PCT concentration and the predicted probability of scarring; the blue line represents the predicted probability derived from the model, while the shaded area reflects the 95% CI. Observed outcomes are marked as red dots, with patients who developed kidney scars (y = 1) clustered across a range of PCT levels; CI: confidence interval; PCT: procalcitonin.

As shown in Figure 4, there was a positive but weak association between PCT concentration and the predicted probability of scarring. The blue line represents the predicted probability derived from the model, while the shaded area reflects the 95% confidence interval (CI), which widens considerably at higher PCT values, possibly due to data sparsity. Observed outcomes are marked as red dots, with patients who developed kidney scars (y = 1) clustered across a range of PCT levels. Although the model suggests a trend of increasing risk with higher PCT values, the overlap between groups and broad CIs suggests limited predictive reliability.

### 3.6. Nomogram

**Figure 5:**
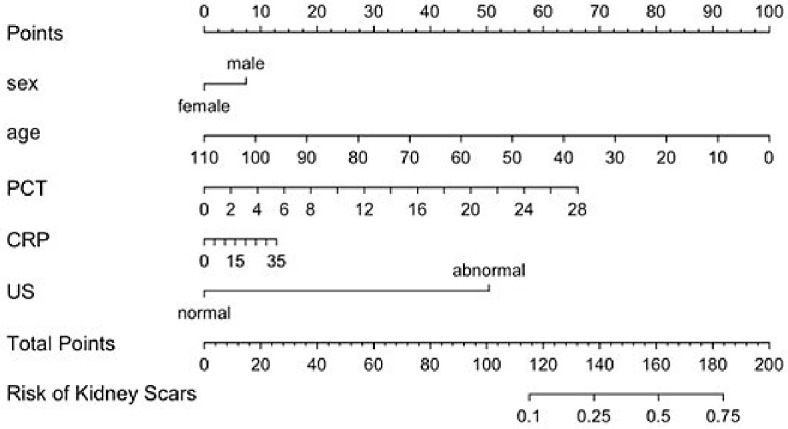
Multivariable logistic regression model predicting the risk of kidney scarring following the first episode of fUTI in pediatric patients (nomogram); CRP: C-reactive protein; fUTI: febrile urinary tract infection; PCT: procalcitonin; US: kidney–ureter–cyst ultrasound.

A nomogram was constructed to visualize the multivariable logistic regression model predicting the risk of kidney scarring following febrile urinary tract infection (Figure 5). The model incorporated five predictors: sex, age, PCT, CRP, and US findings. Each variable is aligned with a point scale at the top of the plot. The sum of these points corresponds to a total score, which is then mapped onto a probability scale at the bottom to estimate the individualized risk of kidney scarring. Among the predictors, abnormal US findings contributed the highest number of points, indicating a strong influence on the predicted risk. Elevated levels of PCT and CRP also increased the predicted probability, though to a lesser extent.

### 3.7. ROC Curve Analysis

**Figure 6:**
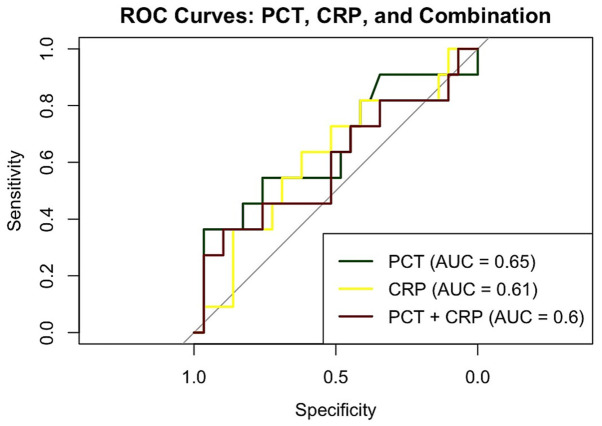
ROC curve analysis assessing the diagnostic performance of PCT, CRP, and their combination in predicting kidney scarring following the first episode of fUTI in pediatric patients; AUC: area under the curve; CRP: C-reactive protein; fUTI: febrile urinary tract infection; PCT: procalcitonin.

ROC curve analysis was performed to assess the diagnostic performance of PCT, CRP, and their combination in predicting kidney scarring following fUTI (Figure 6). The area under the curve (AUC) for PCT was 0.65, indicating modest discriminative ability. C-reactive protein demonstrated slightly lower performance with an AUC of 0.61. Notably, the combination of PCT and CRP did not enhance diagnostic accuracy, yielding an AUC of 0.60. The optimal cut-off value for PCT in predicting kidney scarring was calculated as 6.05 ng/mL, yielding a sensitivity of 36% and a specificity of 97%, while the optimal cut-off value for CRP was 3.62 mg/dL, providing a sensitivity of 63.6% and a specificity of 62.1%.

## 4. Discussion

In children, fUTI, especially acute pyelonephritis, is a leading cause of permanent kidney injury. Approximately 30% of patients with recurrent fUTI will develop kidney scarring [3], and children with VUR are more likely to face recurrent episodes of fUTI and develop kidney scarring [2,3].

In the past, many studies have tried to examine the correlation between inflammatory markers and kidney scarring after an episode of fUTI [18,19,20] or in patients with a history of VUR [21,22,23] in the pediatric population. A recent meta-analysis revealed that PCT and urinary neutrophil gelatinase-associated lipocalin (NGAL) were higher in patients with kidney scarring after an episode of fUTI compared to pediatric patients without scars [10].

Procalcitonin is a propeptide of calcitonin without hormonal activity [24]. It is believed that PCT is produced by the liver and mononuclear cells and is modulated by sepsis-related cytokines in response to bacterial endotoxins [25]. It is almost undetectable under normal conditions or during viral infections. During bacterial infections, PCT increases in ranges from 1 to 1000 ng/mL, and this increase often correlates with the severity of infection. The increase in PCT occurs rapidly after a bacterial infection, especially when compared to CRP levels. Procalcitonin is detected in the plasma two hours after the injection of endotoxins, and a plateau is reached after approximately 12 h, with levels decreasing to normal after two to three days [25,26]. Compared to CRP and WBC, previous systematic reviews and meta-analyses have revealed the superiority of PCT in kidney scar detection after fUTI [27,28]. Furthermore, Zaffanello et al. suggested that children with very high PCT levels during fUTI episodes are likely to be at risk of VUR [28].

In this study, we investigated the association of PCT, CRP, and WBC as markers for predicting kidney scarring after the first episode of fUTI and in the presence of VUR. We also investigated the differences between pediatric patients with a first episode of fUTI with or without VUR and kidney scarring. There were no patients taking antibiotic prophylaxis. No statistically significant differences were detected between individuals with and without VUR and kidney scarring regarding PCT and WBC levels. In contrast, statistical significance was reached in CRP and serum urea levels (*p* = 0.03) between VUR and no-VUR patients with a first episode of fUTI. Additionally, further significant differences between the study groups were noticed. Patients with VUR more frequently had kidney scars after the first episode of fUTI, and that abnormal US findings and VUR were more frequent in individuals who developed scarring after the first episode of fUTI compared to those without scars. Our study suggests that inflammatory markers such as PCT and CRP lacked sufficient predictive value, whether assessed individually or in combination. Abnormal US findings were revealed as the most significant predictor of kidney scarring in both univariate and multivariate analyses. In contrast, severe VUR did not retain statistical significance in the final logistic regression model, despite its positive association with kidney scarring. Finally, the nomogram and ROC analyses further highlighted the limited diagnostic power of PCT and CRP and underscored the need for new biomarkers.

Nevertheless, there are several limitations that should be taken into consideration. This is not a novel study. The relation between PCT, CRP, and WBC and kidney scarring and VUR in the pediatric population after fUTI episodes has already been studied [10]. However, the findings of the present study did not demonstrate statistically significant predictive value for these markers. Several factors may explain this discrepancy. First, meta-analyses aggregate data from heterogeneous populations and study designs, often enhancing statistical power and the detection of effects, whereas our study is a single-center prospective cohort with a limited sample size and a relatively small number of scarring events. Second, our cohort consisted predominantly of young infants with a first episode of fUTI, which may represent a different risk profile compared to mixed or rUTI populations included in previous studies. Real-world clinical settings may have attenuated the observed associations. Therefore, our findings do not necessarily contradict existing literature but rather highlight the challenges of translating biomarker performance into routine clinical practice.

Furthermore, NGAL has shown promising results in previous studies, but it was not included due to logistical limitations; future studies incorporating NGAL and other emerging biomarkers are warranted. Biomarkers such as serum calprotectin have shown promising diagnostic performance in distinguishing bacterial infections in pediatric populations and may represent a useful addition to future biomarker-based risk stratification strategies [29].

Although non-*Escherichia coli* pathogens were observed in a substantial proportion of cases, the sample size within individual pathogen subgroups was insufficient to permit reliable analysis of their association with structural abnormalities or kidney scarring. Larger studies are required to better elucidate the potential role of pathogen-specific risk in renal outcomes following febrile urinary tract infection. Although the cohort predominantly consisted of infants, a small number of older children were included. Given their limited number and the lack of a significant association between age and kidney scarring, their inclusion is unlikely to have materially influenced the results; however, their presence may contribute to a degree of heterogeneity. No statistically significant association between PCT, WBC and kidney scarring or VUR was detected. Due to the limited number of events, interaction or stratified analyses (e.g., according to VUR status) were not performed, as these would likely be underpowered and prone to overfitting. The presence of structural abnormalities, as reflected by acute-phase abnormal ultrasound findings, may confound the interpretation of infection-related scarring. Although the study included only patients with a first episode of fUTI, the prespecified protocol allowed the inclusion of patients with rUTI episodes followed by normal DMSA scans, which may have influenced the study strength by introducing heterogeneity in baseline risk for kidney scarring. Finally, further analysis highlighted the limited diagnostic power of PCT and CRP.

## 5. Conclusions

In conclusion, our study’s results contrast with recent data suggesting that PCT is higher in pediatric patients with kidney scarring after an episode of fUTI compared to those without scars. No statistically significant difference was detected between individuals with and without VUR and kidney scarring regarding PCT and WBC levels. On the contrary, statistical significance was reached in CRP and serum urea levels between the groups with and without VUR. Abnormal US findings and severe VUR were the most significant predictors of kidney scarring. Therefore, the study’s biomarkers demonstrated limited and inconsistent predictive performance, despite PCT’s high specificity at higher thresholds. Further studies with larger study groups, novel biomarkers, and analysis are needed to investigate the possibility of early kidney scar prediction or their correlation with VUR.

## Figures and Tables

**Table 1:** Baseline characteristics of the included patients.

Variables	All Participants (*n* = 65)
Age (months)	4.00 [2.00–8.00]
Sex (male; *n*, %)	37 (56.9)
Fever duration (days)	2.00 [1.00–3.00]
PCT (ng/mL)	0.29 [0.08–1.33]
CRP (mg/dL)	2.71 [0.60–9.86]
WBC (/mm^3^)	16,312.92 (7586.16)
Serum creatinine (mg/dL)	0.35 [0.32–0.40]
Serum urea (mg/dL)	16.00 [14.00–23.00]
Hydronephrosis (*n*, %)	12 (18.5)
VUR (*n*, %)	16 (37.2)
Mild VUR (*n*, %)	2 (4.7)
Moderate VUR (*n*, %)	1 (2.3)
Severe VUR (*n*, %)	13 (30.2)
Abnormal US (*n*, %)	18 (27.7)
Prematurity (*n*, %)	11 (16.9)
Kidney scarring (*n*, %)	13 (28.9)

**Abbreviations**; CRP: C-reactive protein; PCT: procalcitonin; VUR: vesicoureteral reflux; US: ultrasound; WBC: white blood cells. Data are presented as mean (SD), median (IQR) or numbers (*n*, %).

**Table 2:** Comparison of baseline characteristics between included patients with and without VUR; VUR: vesicoureteral reflux.

Variables	fUTI with VUR (*n* = 16)	fUTI Without VUR (*n* = 27)	*p*-Value
Age (months)	4.50 [2.00–8.25]	3.00 [2.00–5.50]	0.56
Sex (male, *n*%)	7 (43.8)	19 (70.4)	0.16
Fever duration (days)	1.00 [1.00–2.00]	1.00 [1.00–2.50]	0.92
PCT (ng/mL)	1.22 [0.21–4.17]	0.24 [0.07–1.29]	0.21
CRP (mg/dL)	8.60 (7.93)	1.19 [0.38–3.96]	***0.03***
WBC (mm^3^)	16,833.75 (7429.40)	15,976.67 (8691.72)	0.74
Serum urea (mg/dL)	22.00 [15.75–26.25]	16.00 [12.00–19.00]	***0.03***
Serum creatinine (mg/dL)	0.39 [0.34–0.45]	0.35 [0.31–0.38]	0.18
Kidney scarring (*n*%)	9 (60.0)	4 (19.0)	***0.03***
Prematurity (*n*%)	2 (12.5)	5 (18.5)	0.70
Abnormal US (*n*%)	8 (50.0)	8 (29.6)	0.31

**Abbreviations**; CRP: C-reactive protein; fUTI: febrile urinary tract infection; PCT: procalcitonin; US: ultrasound; VUR: vesicoureteral reflux; WBC: white blood cells. Data are presented as mean (SD), median (IQR) or numbers (*n*/%).

**Table 3:** Comparison of baseline characteristics between included patients with and without kidney scarring.

Variables	fUTI with Kidney Scarring (*n* = 13)	fUTI Without kidney Scarring (*n* = 32)	*p*-Value
Age (months)	4.00 [2.00–8.00]	4.00 [2.00–7.00]	0.89
Sex (male, *n*%)	9 (69.2)	18 (56.2)	0.64
Fever duration (days)	1.00 [1.00–2.00]	2.00 [1.00–3.25]	0.27
PCT (ng/mL)	2.10 [0.16–6.81]	0.39 [0.05–2.03]	0.15
CRP (mg/dL)	8.62 (8.42)	1.30 [0.30–7.58]	0.14
WBC (mm^3^)	18,285.38 (7012.42)	16,447.81 (8451.64)	0.49
Serum urea (mg/dL)	21.00 [16.00–24.00]	16.00 [12.75–23.50]	0.18
Serum creatinine (mg/dL)	0.38 [0.33–0.40]	0.35 [0.31–0.41]	0.69
VUR (*n*%)	9 (69.2)	6 (26.1)	***0.03***
Prematurity (*n*%)	1 (7.7)	6 (18.8)	0.65
Abnormal US (*n*%)	9 (69.2)	8 (25.0)	***0.008***

**Abbreviations;** CRP: C-reactive protein; fUTI: febrile urinary tract infection; PCT: procalcitonin; US: ultrasound; VUR: vesicoureteral reflux; WBC: white blood cells. Data are presented as mean (SD), median (IQR) or numbers (*n*/%).

**Table 4:** Multivariable logistic regression identifying independent predictors of kidney scarring.

Model	Predictor	Odds Ratio	95% CI (Lower)	95% CI (Upper)	*p*-Value
Full Model	(Intercept)	0.1087187	0.01445873	0.4746048	0.010117580
Full Model	USabnormal	6.9940464	1.23630010	53.5343914	0.037587501
Full Model	VUR_severe	3.7154237	0.63616350	26.3766274	0.154675753
Full Model	PCT	1.0716799	0.90390978	1.2528432	0.345571020
Full Model	CRP	0.9822358	0.87114227	1.0930882	0.744544817
Reduced Model	(Intercept)	0.1057958	0.01421654	0.4529113	0.008637553
Reduced Model	USabnormal	6.3908145	1.22919648	44.1825717	0.036984327
Reduced Model	VUR_severe	3.4304116	0.62564529	21.9084703	0.163207121
Reduced Model	PCT	1.0655121	0.90294176	1.2362015	0.378419735

## Data Availability

The original contributions presented in this study are included in the article. Further inquiries can be directed to the corresponding author(s).

## Abbreviations

AUTH	Aristotle University of Thessaloniki
AUC	area under the curve
CI	confidence interval
CRP	C-reactive protein
DMSA	technetium-99m dimercaptosuccinic acid
ESBL	extended-spectrum beta-lactamases
fUTI	febrile urinary tract infection
NGAL	neutrophil gelatinase-associated lipocalin
OR	odds ratio
PCT	procalcitonin
ROC	receiver operating characteristic
rUTI	recurrent urinary tract infection
US	kidney–ureter–cyst ultrasound
VCUG	voiding cystourethrography
VUR	vesicoureteral reflux
WBC	white blood cells
